# Genetic and demographic consequences of range contraction patterns during biological annihilation

**DOI:** 10.1038/s41598-023-28927-z

**Published:** 2023-01-30

**Authors:** Jordan E. Rogan, Mickey Ray Parker, Zachary B. Hancock, Alexis D. Earl, Erin K. Buchholtz, Kristina Chyn, Jason Martina, Lee A. Fitzgerald

**Affiliations:** 1 Department of Ecology and Conservation Biology (ECCB), Texas A&M University, College Station, USA; 2 Applied Biodiversity Science Program, Texas A&M University, College Station, USA; 3 Ecology & Evolutionary Biology (EEB) PhD Program, Texas A&M University, College Station, USA; 4 Oahu/Maui National Wildlife Refuge Complex, US Fish & Wildlife Service, Hawaii, USA; 5 Present Address: Conservation International, Arlington, VA USA; 6 Present Address: Department of Ecology & Evolutionary Biology, University of Michigan, Ann Arbor, USA; 7 Present Address: Department of Ecology, Evolution, and Environmental Biology, Columbia University, New York, NY USA; 8 Present Address: USGS South Carolina Cooperative Fish & Wildlife Research Unit, Clemson, SC USA; 9 Present Address: Department of Biology, Texas State University, San Marcos, TX USA

**Keywords:** Biodiversity, Conservation biology, Population genetics

## Abstract

Species range contractions both contribute to, and result from, biological annihilation, yet do not receive the same attention as extinctions. Range contractions can lead to marked impacts on populations but are usually characterized only by reduction in extent of range. For effective conservation, it is critical to recognize that not all range contractions are the same. We propose three distinct patterns of range contraction: shrinkage, amputation, and fragmentation. We tested the impact of these patterns on populations of a generalist species using forward-time simulations. All three patterns caused 86–88% reduction in population abundance and significantly increased average relatedness, with differing patterns in declines of nucleotide diversity relative to the contraction pattern. The fragmentation pattern resulted in the strongest effects on post-contraction genetic diversity and structure. Defining and quantifying range contraction patterns and their consequences for Earth’s biodiversity would provide useful and necessary information to combat biological annihilation.

## Introduction

Widespread impoverishment of biodiversity, referred to as “biological annihilation”^1^ is occurring across taxon groups and ecological scales. Focusing attention solely on extinctions underestimates the severity of the biodiversity crisis^2^. Findings on range contraction for mammals by Ceballos et al.^1^ are alarming: nearly all of the 177 species examined have lost 40% or more of their geographic range, with almost half losing more than 80%. In a separate analysis, Ceballos et al.^3^ found that for 48 mammal and 29 bird species on the brink of extinction, there was an estimated reduction of 95% and 94% in their ranges since 1900, respectively. While the gravity of species extinctions is a compelling narrative within the biodiversity crisis, extinction accounts for only a small portion of overall biodiversity decline^3,4^.

Range contraction is typically described as the amount of range lost^1,3^. However, range contractions can take various patterns beyond the spatial extent of loss and each of these patterns can have different consequences for species’ populations. Range contractions in general are large-scale disturbances that deplete populations and reduce genetic diversity by altering spatial configuration and amount of suitable habitat^5–8^. Effects of specific patterns of range contraction on population demography and genetics are understudied. The spatial impact of different patterns of range contraction will vary according to the contraction pattern, and these impacts may be observed well into the future. Indeed, Branco et al.^8^ found that Paleolithic range contraction influenced the modern-day spatial gradient of human genetic diversity. Different patterns may necessitate different conservation strategies, such as conserving disjunct populations, prioritizing conservation corridors, reintroduction planning, or restoring habitat within a species’ historic range.

Two general patterns are discussed in the literature on range contractions—contraction to the range core and contraction to the range periphery. Evidence suggests that ranges most often contract toward their peripheries^9–13^, but local, regional and historical factors can create exceptions^12,14,15^.

Genetic and demographic factors are key determinants of population viability and species survival^16^. Declining populations of many threatened or endangered species have been shown to suffer from a complex synergy of genetic and demographic consequences ultimately resulting from demographic depletion, consequent inbreeding and overall reduced fitness^17–19^. The influence of anthropogenic disturbance on genetic and demographic components of populations has been addressed by examining disruptions in parameters such as nucleotide diversity^20^, relatedness^18^, age structure^21,22^, number of offspring^23^, and reproductive fitness^19^ as these parameters have been found to be crucial factors in determining the persistence of populations^16,18^.

Genetic diversity has emerged as an important baseline measure of population health and viability in the field of conservation genetics^24–26^. This is due to two key principles of genetic diversity. Firstly, neutral diversity for a diploid species is a product of the effective population size and the mutation rate (*θ* = 4*N*_*e*_*μ*); therefore, given some constant mutation rate, we can straightforwardly interpret neutral diversity as informative about the effective population size. Secondly, adaptive and deleterious diversity do not have as obvious a relationship; instead, these variants rely on the inequality 1 ≤|4*N*_*e*_*s*|, where *s* is the selection coefficient^27^. When |4*N*_*e*_*s*| is greater than 1, selection is expected to dictate the frequency of a given allele; when less than 1, drift is the dominant force. Since selection can take on a myriad of forms and is often context dependent, it is not easy to interpret results from simulations studies that incorporate selective variation due to the reliance on arbitrary values of *s*. Furthermore, the distribution of fitness effects (DFE) observed in most natural systems demonstrates that adaptive variation represents a small fraction of the standing variation, with most being neutral or nearly neutral^28^). Therefore, measures of neutral genetic diversity represent the most straightforward technique for assessing population sizes in most natural systems. However, to our knowledge, the potential consequences of distinct patterns of range contraction on genetic diversity of species’ populations have not been explored so far, particularly, how different conformations of range contractions may decouple the expected relationship between genetic diversity and population size^27^.

**Figure 1:**
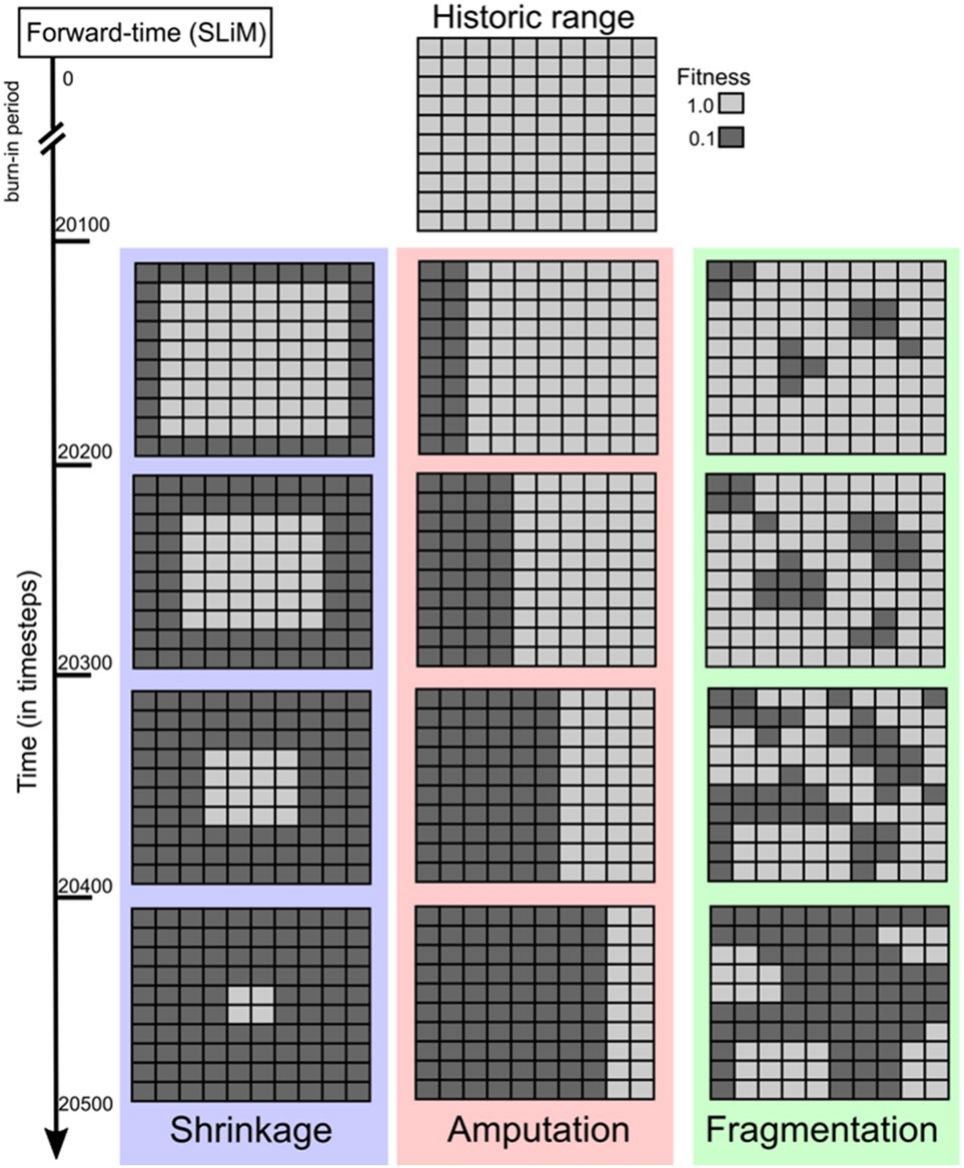
Representation of the simulation of three patterns of range contraction: amputation, shrinkage, and fragmentation. The historic range is represented at the top of the figure, with light gray squares representing habitat fitness of 1.0. Below are the three range contraction scenarios, with the darker grey squares representing fitness values of 0.1. The y-axis is time (in simulation time-steps), with each contraction occurring in a discrete interval. The landscapes are here shown as grids for simplicity, but note fitnesses are interpolated across cells for smooth transitions.

Recent advances in simulation software^29–31^ have expanded our ability to assess how range contractions impact populations. We consider here three patterns of range contraction: shrinkage, amputation, and fragmentation (Fig. 1*)****.*** Amputation occurs when reduction of geographic range begins at a point in the periphery of the species’ range and spreads across the range until the last remaining populations occur in areas that are furthest from the initial population extinctions. In short, portions of the species’ range are amputated with the advancing extinction front. Shrinkage describes the scenario where a species’ range contracts from its periphery to its core. This pattern has also been referred to as a “melting range”^32^ or “range collapse”^33^. Fragmentation at the scale of species’ ranges occurs through land cover changes that create disjunct populations because of loss of continuity in the range. Range fragmentation constrains dispersal, which may impact genetic diversity. Range fragmentation and habitat fragmentation are distinct but not mutually exclusive. Habitat fragmentation affects the spatial configuration of habitat used by a species at local scales and can occur whether or not the species’ range is reduced. The genetic consequences of habitat fragmentation have been well-documented in the literature. Consequences can include reductions in heterozygosity and allelic diversity and richness^34^, altered distribution of genetic diversity^7^, increased genetic isolation^35^, and increased inbreeding^36^. In this study, we seek to improve our understanding of fragmentation at the scale of the range and thus consider range fragmentation a form of range contraction. Range fragmentation results in a smaller total area available to be occupied by a species, though the geographic extent of the range is similar to its historical baseline. Fragmentation of species’ ranges occur due to conversion of land cover types at the range-scale. These three patterns of range contraction can be deduced when contractions are recent and historical range data exists. These patterns of range contractions have been observed in a wide variety of taxa worldwide and have been attributed to a number of different drivers (Table 1).

**Table 1:** Examples from the literature of range contractions in various taxa representing three patterns (amputation, shrinkage, and fragmentation), along with potential drivers of each contraction, as described by the authors of each study.

Taxonomic group	Species	References	Contraction pattern	Driver(s)
Anthozoa	Coral communities	^76^	Amputation	Climate change
Birds	Bachman's Warbler (*Vermivora bachmanii*)	^77^	Fragmentation	Habitat destruction
Birds	Pampas Meadowlark (*Sturnella delfilippii*)	^78^	Shrinkage	Habitat destruction
Herpetofauna	Eastern Massasauga Rattlesnake (*Sistrurus catenatus*)	^79^	Amputation	Climate change, land cover
Herpetofauna	New Zealand herpetofauna	^80^	Amputation	Introduced mammals
Herpetofauna	Louisiana Pine Snake (*Pituophis ruthveni*)	^81^	Fragmentation	Habitat destruction
Herpetofauna	Blanchard's Cricket Frog (*Acris blanchardi*)	^82^	Amputation	Water contamination
Herpetofauna	Cascades Frog (*Rana cascadae*)	^83^	Amputation	Invasive species, habitat destruction
Insects	Butterflies	^84^	Amputation	Climate change
Mammals	American Pika (*Ochotona princeps*)	^85^	Fragmentation	Climate change
Mammals	Iberian Lynx (*Lynx pardinus*)	^86^	Fragmentation	Prey abundance, land use changes
Mammals	Spectacled Bear (*Tremarctos ornatus*)	^87^	Fragmentation	Habitat destruction
Mollusks	Blue mussel (*Mytilus edulis*)	^88^	Amputation	Climate change
Plants	*Scythothalia dorycarpa*	^89^	Amputation	Marine heat wave

We hypothesized that distinct contraction patterns would produce different demographic and genetic consequences, and these consequences would not be uniform throughout post-contraction ranges. We investigated this hypothesis by simulating the demographic and genetic effects of three different range contraction patterns on a generalist species in combination with the spatial locations of individuals. We evaluated the significance of these impacts on range-wide population diversity, and made recommendations for mitigating future species’ declines due to range contractions.

Our goal was to gain insights into the interplay between range contraction and its consequences for genetic diversity and demography, according to the three patterns of range contraction defined above. In addition to tracking nucleotide diversity, we also mapped spatial ancestry, which has been shown to be impacted by range contractions^5^ Spatial ancestry combines the geographic locations of individual ancestors in the past, as well as their relative genomic contribution to any individual living in the present. From this information, we can evaluate how patterns of range contractions bias the distribution of ancestors backward in time. Classical spatial genetics predicts that spatial autocorrelation in relatedness should decay as ancestors spread out across the range, eventually losing any signature of the geographic location of present-day descendants^37^. However, demographic disequilibrium is expected to skew the rate at which this transition occurs, and patterns of spatial ancestry may become biased in the direction of the range contraction. In terms of conservation, this would have implications for restoration and translocation efforts due to the potential loss of locally adapted alleles. In addition, such a pattern would hamper our ability to accurately estimate dispersal distances, as individuals that persisted into the present may be descendants of individuals who moved very little (if occupying an area not affected by contraction) or very far (if originally within the pre-contraction range).

Finally, we examined how sampling individuals from different parts of the range can alter interpretation of the impacts of range contraction. Our models allow us to make generalizable predictions about the magnitude and timing of effects of range contraction on genetic diversity and demography, and how the spatial distribution of ancestry and genetic diversity influence the interpretation of these effects. These insights can serve to inform the development of conservation interventions aimed at confronting the challenge of biological annihilation.

## Results

**Figure 2:**
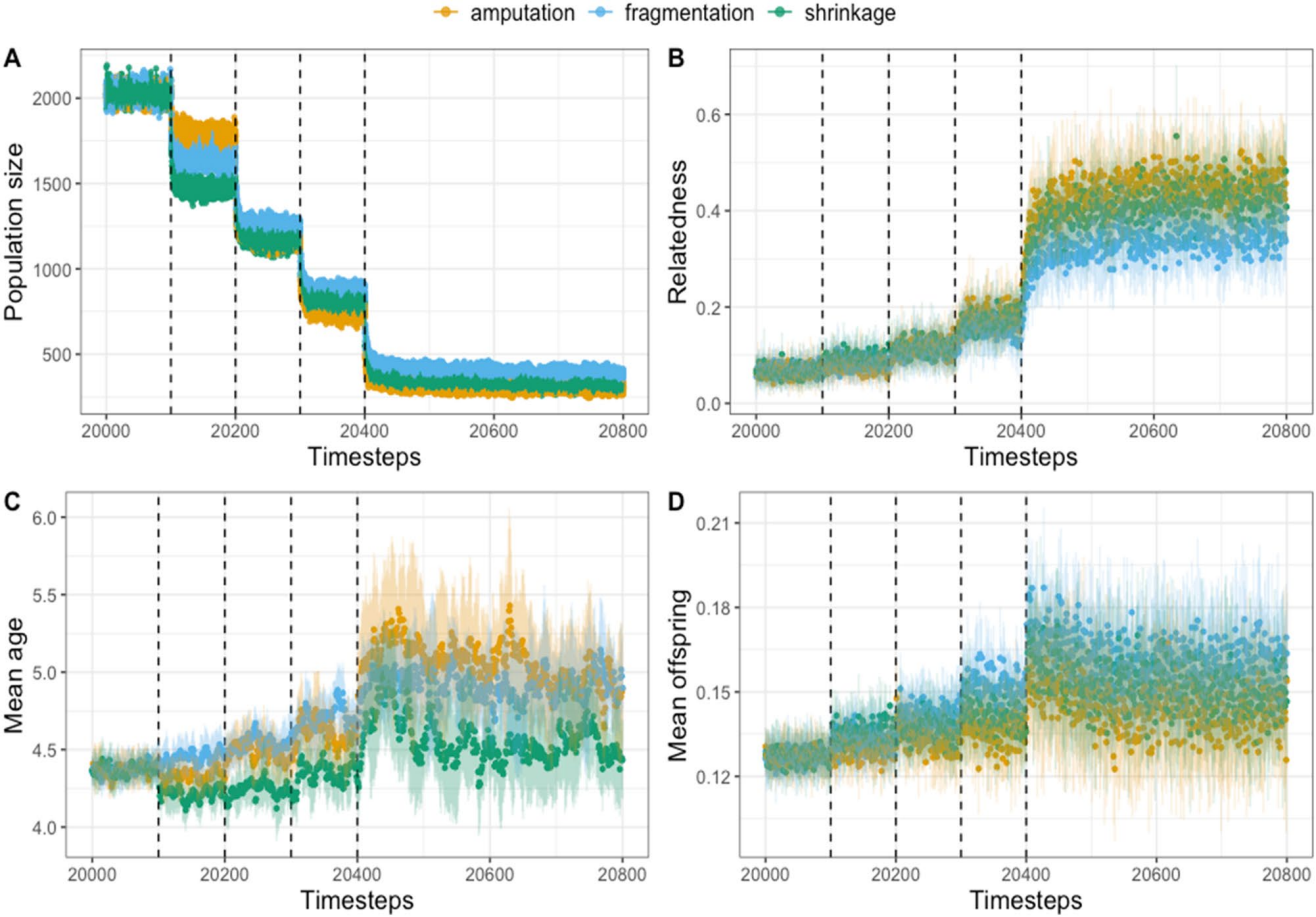
Effects of three simulated patterns of range contraction (amputation, fragmentation, and shrinkage) on (**a**) population size following each discrete range contraction event; (**b**) pedigree relatedness across timesteps following contraction; (**c**) mean age of individuals in the population following range contraction; (**d**) mean number of offspring across timesteps for each replicate. Dashed lines represent the timing of each discrete range contraction event; each point is the mean and lines are the standard deviation of 10 replicates.

**Figure 3:**
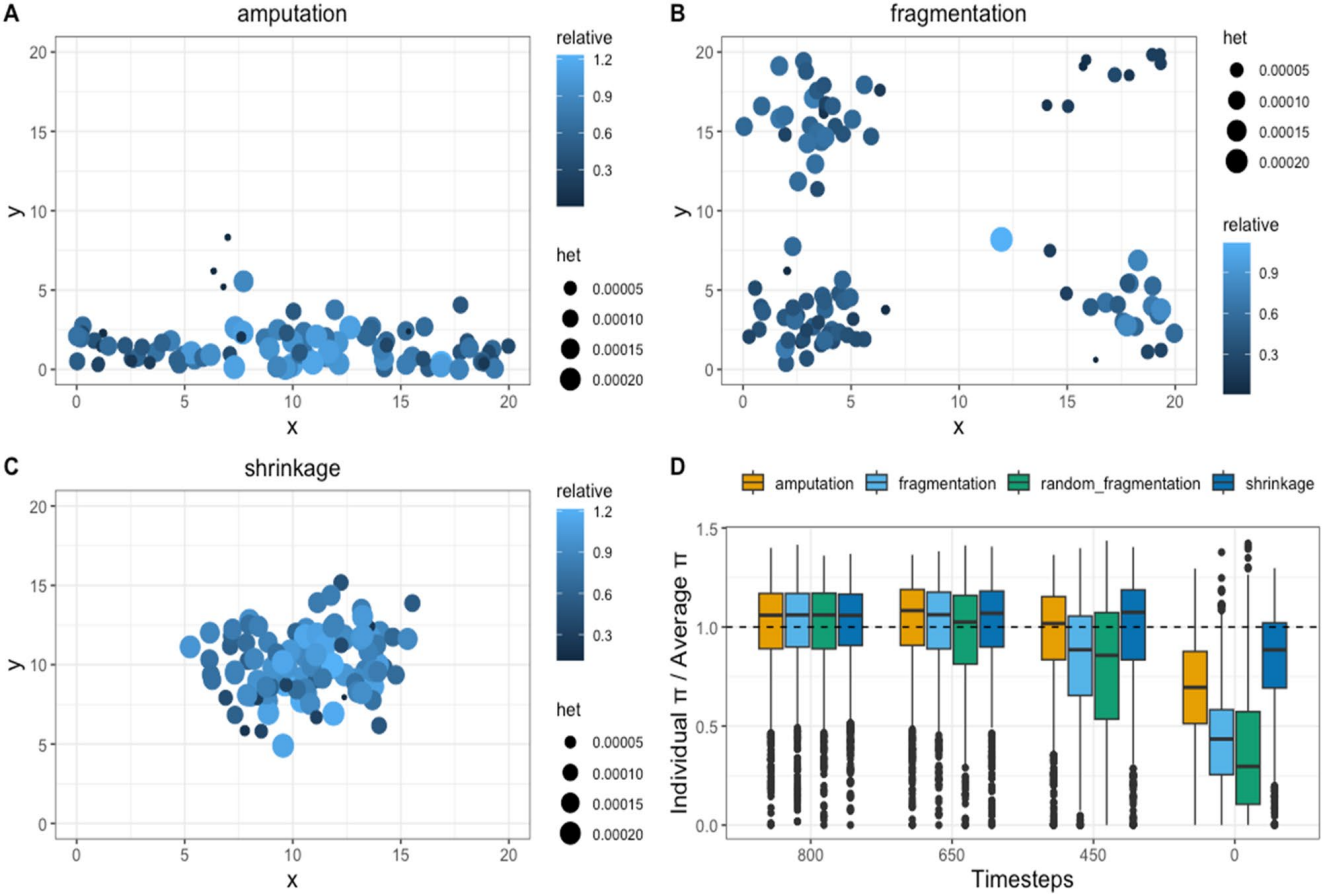
Spatial distribution of individual post-contraction nucleotide diversity (π) for three range contraction patterns from a representative replicate: (**A**) amputation; (**B**) fragmentation; (**C**) shrinkage. Circles represent individual locations, size of the circles are individual nucleotide diversity, and color is individual nucleotide diversity relative to the pre-contraction population mean. (**D**) Change in individual diversity relative to the pre-contraction mean across all ten replicates, including the random fragmentation model, with the dashed line representing the pre-contraction mean.

Simulated range contraction models resulted in population declines of 86–88%, (Fig. 2A, Table S1). All models showed significant increases in average relatedness (*p* < 0.001 in all cases; *r*^2^ ranged from 0.33 to 0.65; Fig. 2B), but the slope of the relationship between relatedness and timesteps was steepest in the amputation scenario. Variability in relatedness increased following range contraction (Fig. 2B). By 400 timesteps after the contraction (timestep 20,800), all patterns displayed significant decreases in mean nucleotide diversity relative to pre-contraction diversity, with fragmentation suffering the largest decrease of > 50% of its pre-contraction diversity. In addition, nucleotide diversity was strongly influenced by spatial location, with some models maintaining regions of high diversity comparable to pre-contraction levels (Fig. 3A–C). While each model showed an eventual decline in nucleotide diversity, they differed in the number of generations before nucleotide diversity became significantly less than pre-contraction conditions (Fig. 3D). By 50 timesteps after the end of the contraction (timestep 20,450), both amputation and fragmentation had fallen below the pre-contraction mean nucleotide diversity, while shrinkage had not yet shown a significant decrease. For all models, the average and max age of individuals increased as species’ ranges contracted (*p* < 0.001; Fig. 2C,D). Finally, the mean number of offspring appeared to initially increase during the contraction, but 400 timesteps later had begun to trend downward (Fig. 2D).

**Figure 4:**
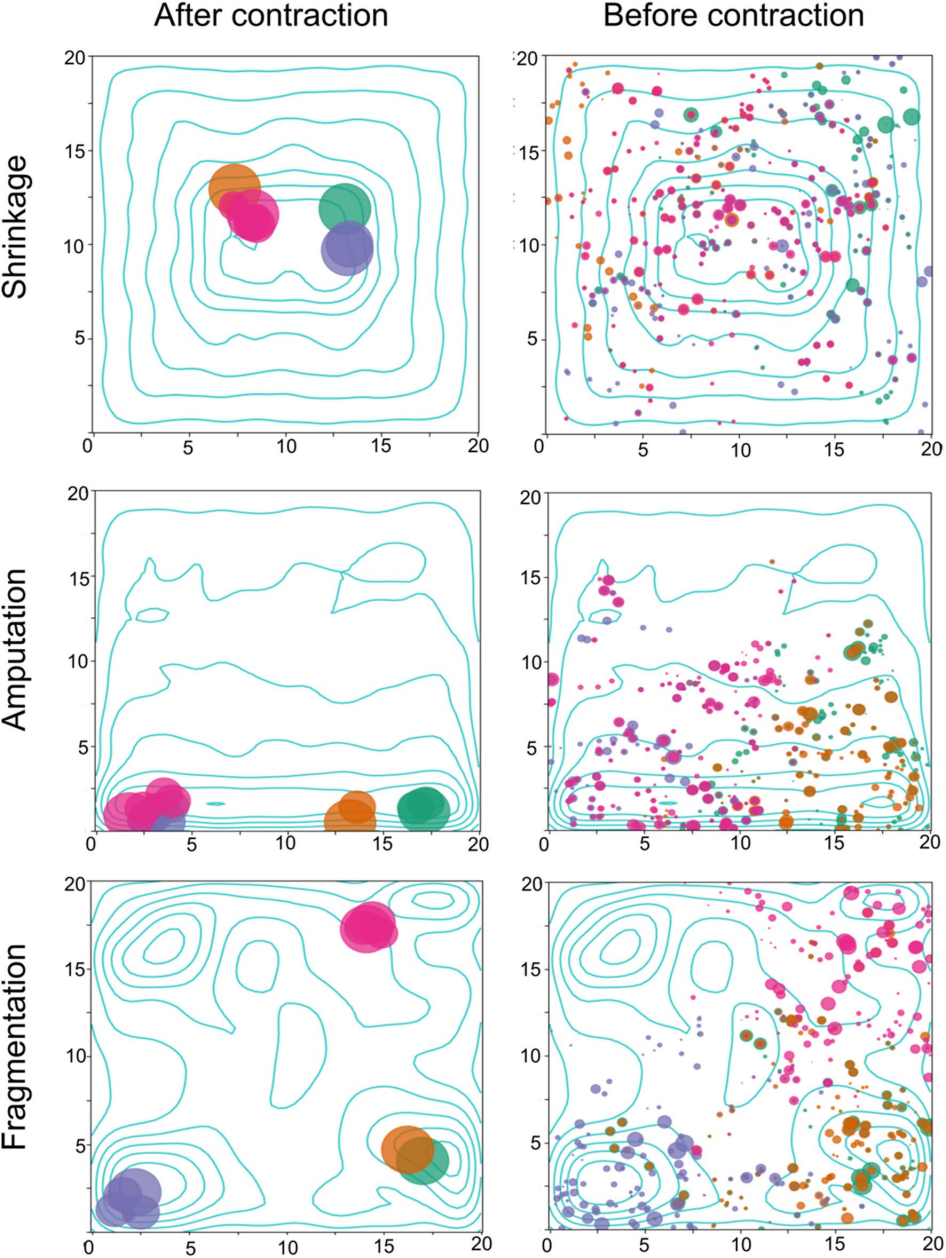
The spread of spatial ancestry of 4 randomly sampled individuals in the post-contraction range for each of the three simulated patterns of range contraction (shrinkage, amputation, and fragmentation) from a representative replicate. Colors represent the focal individual, and the size of the circle is the proportion of genomic contribution from a given ancestor. Multiple circles appear in the sampled time-slice due to overlapping generations (i.e., parents and grand-parents are still present). “After Contraction” samples were taken 50 timesteps after the last discrete contraction interval, whereas “Before Contraction” were taken 50 prior to the initial contraction. Contour lines represent population density at the end of the simulation.

The shrinkage model was the most resilient to range contraction, with individuals in the center of the range maintaining diversity near or equal to pre-contraction nucleotide diversity (Fig. 3C). A pseudo-edge effect is apparent by 400 timesteps after the contraction, in which individuals nearest the contracting edge display the lowest individual nucleotide diversity. Due to this maintenance of diversity in the core of the range, shrinkage maintained pairwise divergences (π_12_) between sampled groups that were similar those in the other contraction scenarios, despite having lower *F*_ST_ (Tables S2 and S3). No pattern of isolation-by-distance was apparent in the shrinkage model. The spatial spread of ancestry for the shrinkage model was the only contraction scenario that resulted in ancestors roughly distributed randomly across the range 50 timesteps prior to the contraction (Fig. 4). This pattern is expected given the high levels of diversity maintained in the shrinkage model; the core of the range consists of individual migrants from each corner of the landscape, each of which introduce their unique geographic variants that may be locally lost over time to drift.

The amputation model was intermediate between shrinkage and fragmentation with respect to its impact on average nucleotide diversity, losing ~ 25% of its diversity prior to pre-contraction conditions (Fig. 3D). As with shrinkage, amputation maintained relatively high diversity near the center of its post-contraction range, with individuals nearest the corners of the range having the lowest absolute and relative individual nucleotide diversity (Fig. 3A). This is also reflected in the average nucleotide diversity of groups sampled at the edges of the post-contraction range (Table S4). Amputation produced strong patterns of isolation-by-distance, with *F*_ST_ being the highest between groups sampled at the opposite ends of the range (*F*_ST_ = 0.3871). Spatial ancestry in the amputation model was strongly biased towards the extinction front, with very few ancestors from the upper half of the range living 50 timesteps before the contraction.

The fragmentation scenario produced the largest loss in average nucleotide diversity relative to pre-contraction conditions, suffering a > 50% decline in diversity (Fig. 3D). Few individuals possessed heterozygosity comparable to the pre-contraction population mean, with the highest concentrated in the two larger pockets of habitable area (Fig. 3B). Despite these trends, fragmentation had the lowest increase in mean relatedness of the three scenarios, likely reflecting the lack of connectivity between surviving demes. Fragmentation also initially had the highest increase in mean number of offspring during the contraction. Like amputation, the fragmentation model showed strongly biased spatial ancestry, with clouds of ancestors clustered around the focal individuals sampled location persisting even 50 timesteps prior to the contraction. Our specific fragmentation configuration had little effect on our results, as they were qualitatively like a randomly generated fragmentation scenario (Fig. S5).

## Discussion

Our results demonstrated how range contractions can contribute to biological annihilation not only through loss of area inhabited by a generalist species, but also due to impacts on demography and loss of genetic diversity. Our simulations revealed that the extent and magnitude of effects differed depending on the pattern of range contraction. The unique outcomes resulting from amputation, shrinkage, and fragmentation underscore the importance of documenting how range contractions occur in real-world ecosystems. Range contraction can take many forms. Our models are an important step towards a general understanding of what impacts are likely to manifest under different range contraction patterns. This is crucial when considering conservation strategies for preservation or recovery of populations.

### Genetic and demographic consequences of range contraction patterns

The sensitivity of nucleotide diversity to reductions in population size has been a topic of debate for some time^38,39^, particularly whether it responds to population reductions within the timescale of relevance to anthropogenic causes of range contractions. Concordant with previous studies^40,41^, we found that average relatedness responded much more rapidly to reductions in absolute population sizes than nucleotide diversity for our simulated generalist species. This occurred in all three range contraction patterns. In the most extreme case, shrinkage, we found that a decline in nucleotide diversity may not be detectable until > 400 timesteps after range contraction despite a > 85% loss in range area (Fig. 3). This finding demonstrates a pressing need for the field of conservation genetics to adopt more sensitive measures of population health than nucleotide diversity. Blanco-Pastor et al.^42^ predicted that genetic diversity of an endemic alpine plant will not experience drastic decreases with severe range contractions. However,^43^ leveraged SNP data on Florida scrub-jays with a full population pedigree and gene-dropping simulations tracked shifts in allele frequency dynamics in only a few generations. This study is particularly interesting in the context of our spatial ancestry spread analyses; for example, with a full population pedigree it would be possible to identify regions of the landscape in which ancestors are never or rarely found. Identifying features of the landscape that correspond to reduced ancestry presence may aid conservation biologists in pinpointing the historic causes of range contraction. Recently^44^, attempted to quantify the extent of loss of diversity across multiple plant and animal species by utilizing segregating sites instead of pairwise nucleotide diversity. Unfortunately, these techniques require either thorough population pedigrees or deep genomic coverage.

We demonstrated the rate of decline of nucleotide diversity within a population was highly impacted by the spatial pattern of contraction, mirroring the results from the spatial ancestry spread analyses. Contraction patterns that maintained high connectivity and impacted the periphery of the range most heavily (such as shrinkage) tended to be resilient to declines in nucleotide diversity. Because population density was highest in the core of the range, the loss of peripheral individuals did not remove the bulk of standing diversity^37^. As expected, amputation, which constrained the remaining range towards the edges caused appreciable reductions in standing diversity despite maintaining absolute population sizes similar to those in the shrinkage scenario. Furthermore, the loss of connectivity in fragmentation had dramatic impacts on the rate of decline of nucleotide diversity. Reduced connectivity has been recognized as an important driver of extinction risk of populations^45,46^ .

Discrete sampling in continuous populations is known to bias measures of dispersal and connectivity^47–49^. This is partially due to the metrics of gene flow (such as *F*_ST_) being derived for discrete populations. Furthermore, incomplete sampling across the range may skew the interpretation of the impact of a contraction on measures of diversity. In our simulations, we found that samples taken from the center of the range consistently had higher nucleotide diversity and lower differentiation than those from the edges (Tables S2–S7). Indeed, for the shrinkage pattern, the level of nucleotide diversity in the range core was comparable to the pre-contraction conditions long after the contraction ended. This demonstrates the importance of having prior knowledge about range size and boundaries and patterns of occupancy throughout the range. Our generalist species could occur anywhere in the remaining range, and future investigations could explore how uneven occupancy could influence the results we obtained.

Range contractions also contribute to biological annihilation by altering demographics of populations. Indeed, some alterations in demographic patterns are expected to become apparent following shifts in absolute population size. For example, the age structure of a population may shift towards older age classes following population declines, which has been attributed to reduced survival of juveniles or reproductive failure^21,50^. Our models produced the same trends (Fig. 2C). However, our models have no age-specific fitness declines; instead, these trends occurred due to the increasing threat of dispersing out of the remaining range and dying. Since only juveniles dispersed in our models, adults were relatively safe assuming they were not on the contracting edge. In addition, reduced population density relaxed competition between individuals, allowing them to persist for longer. Similarly, in the early stages of range contraction, our models showed a net increase in the mean number of offspring; individuals were living longer and having more offspring. While the mean age continued to increase, the mean number of offspring reached a plateau and, at least in amputation and fragmentation, began to trend downward. However, it is important to note the high variance in means among generations, as well as the means among scenarios (Fig. 2). This increase in variance with subsequent population decline is both statistical and biological—the decreased population size following the contraction is akin to increased sampling error of the population mean. The high variance could potentially make demographic implications of each scenario harder to predict in real-world settings. We recommend that future investigations into the age structure of declining populations account for reduced intraspecific competition as a potential driver of longevity, in addition to the reduced survivability of juveniles. This could include an experimental or observational approach that leads to mechanistic causes of shifts.

### Implications for theories of geographic range

Channell and Lomolino^11,12^ found that with few exceptions, ranges were far more likely to contract to their peripheries (e.g., amputation) than to their cores (e.g., shrinkage). As such, our generalizations for the amputation pattern will likely be the most broadly applicable in natural systems. Though amputation may be the more common pattern of range contraction in natural systems, our findings reveal that individuals on the periphery of the range will be differentially impacted depending on the way an extinction factor spreads. Given that the importance of the range periphery relative to range core for species persistence has been contested in the literature^11,12,32,51^, we can expect that impacts on species experiencing range contraction to the periphery will likely vary according to how extinction factors spread across the range^14^. It is also important to consider that the “abundant center” hypothesis, or the assumption that population density is higher in the center of their range and decreases towards the range edges^11,12,52–54^ has had equivocal support in the literature^14,31,55^. Similarly, it has been suggested that the distribution of genetic diversity in a species’ range prior to contraction may also be non-random and vary considerably between species’ ranges due to factors such as historic demographic processes^56^. If for example, the genetic diversity of a temperate species is concentrated at range edge due to post-glacial expansion, a pattern such as amputation could have a catastrophic impact on this species’ diversity if the highly diverse range edge is eliminated. The pre-contraction distribution of both individuals and genetic diversity throughout a species’ range therefore present critically important implications for the anticipated impacts of different range contraction patterns on species’ populations and deserve careful consideration when evaluating contraction effects.

We chose range contraction patterns that reflect predominant hypotheses in range theory^11,12^. These patterns have been shown to be influenced by local and regional factors, especially history of anthropogenic land use^14,15,57^. It is important to consider patterns of historical range loss when examining effects of range contractions. We are unaware of any published examples of two range contraction patterns occurring concurrently, but it is plausible that different forms of range contraction can take place across a species’ range over time. For example, a range could undergo amputation, then shrinkage. Though we did not simulate successive patterns of range contraction, our results lend insights into how histories of range contraction may affect demography and genetic diversity. We showed that genetic diversity was maintained near pre-contraction levels after shrinkage; however, pre-contraction diversity may not remain in a range that had historically been amputated prior to shrinking. Indeed, Donald and Greenwood^14^ hypothesized that this exact contraction scenario occurred in the British range of the Corncrake (*Crex crex*). Vandergast et al.^35^ found that both past and present fragmentation contributed to population genetic structure of Jerusalem crickets (*Stenopelmatus* ‘*mahogani*’**).**

Fragmentation is a ubiquitous and challenging form of range contraction and biological annihilation^58^, yet, despite the large literature on habitat fragmentation, it has not been adequately addressed in the range contraction literature. Our simulations of range contraction by fragmentation resulted in more drastic effects on genetic diversity and post-contraction population genetic structure than the other patterns. This is perhaps unsurprising due to the well-documented effects of habitat fragmentation on genetic diversity and structure^7,34–36,59^. Range fragmentation can occur naturally over geologic time scales yet is also caused by human land use over rapid time scales^46^. Range fragmentation has also been shown to cause striking demographic disruption^60,61^ that in some instances has directly led to population extinction^22,62^. The majority of fragmentation research is directed at understanding effects of habitat fragmentation on populations^63,64^. Habitat fragmentation may or may not accompany range contraction, especially for a generalist species like we modeled. Including fragmentation in geographic range theory with the other commonly studied patterns (i.e., the contagion vs. demographic hypotheses of Channell and Lomolino^11,12^) is especially relevant considering that land use is a driver of range contraction^15^. We suggest that fragmentation merits further consideration as an important pattern of range contraction across the globe.

### Future research and implications for conservation

The principal implication of our results is that a “one size fits all” conservation approach will not be effective in identifying and ameliorating the consequences of range contraction. We showed fragmentation caused strong genetic differentiation among disjunct range fragments (*F*_ST_ > 0.49 for all comparisons), which resulted in increased pedigree relatedness within isolated groups and decreased genetic diversity relative to other patterns. In natural systems, it may be a priority to develop corridors between fragments to restore gene flow or employ reciprocal introductions to mitigate loss of diversity among remnant populations^65^. Reintroductions may be an important strategy for the amputation scenario, in which connectivity remained high in the remaining range but genetic diversity was low due to the persistence of historically less diverse lineages. Undoubtedly, a complex synergy of unique factors including life history, phylogeny, social group structure, behavioral flexibility, ecological niche, or local and regional factors^15,66–68^ should be considered when developing strategies to combat biological annihilation. While our simulations provide important conservation implications for addressing the impacts of range contraction on species’ populations, we acknowledge that the conservation measures we suggest based on our findings may be costly and difficult to implement in practice.

Our finding that the spatial distribution of ancestors was strongly skewed in the direction of the contraction for almost all patterns bears important implications for interventions aimed at addressing the loss of locally adapted gene complexes. This implies that local adaptations may be lost because lineages carrying those adaptations go extinct as the range contracts. This local extinction is dependent on the average dispersal distance, range size, and the rate of the contraction in ways that are beyond the scope of this paper. In general, however, we note that attempts at repatriation in the historic range may be hindered by lack of locally adapted gene complexes, and conservation interventions should be designed to monitor and prevent loss of local lineages^69^. Spatial ancestry was most skewed in amputation*,* what is possibly the most common form of contraction in empirical systems.

We considered several limitations to our simulation model. First, individuals in our models are hermaphroditic, which alleviates the issue of Allee effects. Thus, our results represent a conservative measure of the impacts of range contraction. Future work might consider modelling separate sexes, heterogenous habitat, habitat selection, and complex mating systems. Second, despite living for several generations, individuals only dispersed once immediately after birth, which limited their ability to respond to range contractions. For highly vagile organisms that may reproduce in several locations over their lifetimes, our results would be exaggerated. This limitation can also be mitigated by future work incorporating adult movement following offspring generation, which would allow a greater number of individuals to “escape” the contracting portion of the range. Our simulated individuals were also capable of traversing their entire range in only a few generations, making them highly dispersive relative to some natural populations. We chose this level of dispersion as a conservative estimate, as less dispersive species would show even stronger patterns of spatial ancestry and loss of diversity. Thirdly, our simulated ranges are uniform in their pre-contraction suitability, whereas natural ranges are typically patchier. We also assumed that contraction happens in discrete intervals instead of continuously. We do this for model simplicity, but we recognize that some contractions may happen continuously. In addition, our model did not include selection, which in nature may allow individuals in contracting parts of the range to adapt to their new environment. Finally, we constrained all interaction distances (dispersal, mate choice, and competition) to be identical, but in nature these may differ dramatically. For example, individuals may choose mates from a relatively small area, but then disperse exceptionally far from their place of birth. While varying these parameters can generate stronger or weaker trends, we contend the benefit of our models is in their generality. They create a baseline expectation for how patterns of contraction will differentially impact species.

Empirical studies that explicitly address range contraction patterns are of increasing value to conservation, especially if genetic and demographic correlates are also measured. Patterns of range contraction have typically only been considered in multi-species analyses and reviews^13^, while most reports of range contractions for single species focus on the amount and extent of range lost^70^. Our results show an important next step will be to investigate consequences of contraction patterns in real ecological systems. Understanding range contraction patterns and their consequences for the planet’s biodiversity is crucial to further combat biological annihilation in the Anthropocene.

## Methods

### Population model

Range contraction patterns were modelled using individual-based simulations in SLiM v3.3^31^ (Fig. 1). Ranges were modelled in a continuous-space, 20 × 20 grid with bilinear interpolation to allow smooth transitions of grid-specific fitness effects. At the beginning of the simulation, individuals were distributed uniformly across the range. Contractions occurred in the models by decreasing the fitness of individuals occupying grids within the contracting portion of the range to 0.1 (see Fig. 1). For each of the three range contraction patterns, we ran simulations for 20,000 generations prior to contractions to allow adequate model burn-in. Following the burn-in, 22% of the range was forced to contract in four discrete intervals 100 timesteps apart (Fig. 1) resulting in in ~ 88% total range loss. Genetic diversity theoretically scales with population size (π = 4*N*_*e*_*μ*). To ensure we could conclude that differences observed in reduction of nucleotide diversity were due to the pattern of range contraction and not a result of population decline, we constructed simulations such that population decline was equal among scenarios and co-occurred with a similar amount of range reduction (this was roughly equal among contraction scenarios). If population decline were the driver of reductions in nucleotide diversity, then each scenario would result in equivalent levels of genetic diversity. To ensure that our fragmentation model was not influenced by our specific configuration choice, we also ran a model in which the landscape contracts at random. Simulations continued for another 400 timesteps after the final contraction and were performed ten times per contraction scenario, representing ten completely replicated populations for each scenario.

Our simulations apply to an ecological generalist species with broad habitat requirements. The simulated species does not represent a particular taxon; rather, we chose traits that would make our simulations robust to a wide range of life history strategies. In addition, the specific suite of parameter values we chose guaranteed (1) spatial structure and patterns of isolation-by-distance; (2) dispersal distances that allowed a lineage to traverse the range in at least 40 timesteps (~ 10 generations); and (3) population sizes could be equivalent across contraction scenarios, regardless of final range conformation. In some cases, altering dispersal distance or carrying-capacity resulted in population sizes being unequal following contraction, which would make these scenarios cease to be comparable. Thus, we settled on single parameter values for dispersal, carrying-capacity, and interaction distances. We followed the overall design and concept of Battey et al.^49^ for our model runs. Battey et al.^49^ evaluated the influence of a wide range of parameter values in SliM onWright’s neighborhood size, estimation of genetic diversity, and other summary statistics pertaining to population genetic analysis. We simulated a species with overlapping generations, density-dependent competition, no habitat selection within its range, and spatially explicit mating. For both mate choice and intra-specific competition, distances between individuals were converted into interaction strengths and defined by a Gaussian kernel with a maximum (*m*) of 1 / 2 π σ^2^, where π is the mathematical constant (rather than nucleotide diversity) and σ is the dispersal distance. The interaction strength had a maximum distance of 3σ, beyond which spatial competition and probability of mating are both effectively zero. Each cell of the landscape had a carrying-capacity (*K*) of 5 individuals. For simplicity, individuals were modelled as hermaphroditic but self-incompatible. The number of offspring from each mating pair were chosen based on a random draw from a Poisson distribution with λ = 1/*L*, where *L* (= 4) is the mean age (in timesteps) of individuals within the population at any given generation. Following classical spatial population genetic models^71,72^, offspring dispersed according to a random draw from a normal distribution with a mean of zero and standard deviation σ. Range boundaries are absorbing such that any individuals that disperse outside the range die. Preliminary simulations showed little difference between absorbing and reflecting boundaries; the former decreases fitness by reducing offspring number, the latter reduces fitness by increasing spatial competition. Individual fitness (*W*) of individual *i* can ultimately be defined as a combination of competition and site-specific effects (*h*):

$$W_{i} = \frac{1}{{1 + \frac{\rho m}{h}}},$$

where ρ = λ/[(1 + λ)*K*] and represents the spatial competition constant^49^.

To address the effects of range edges on individual fitness, we corrected the area of the interaction circle for individuals near the edge by recalculating it to represent actual occupiable space (i.e., excluding area of the interaction radius that may fall outside the range). Next, we adjusted the strength of spatial competition to the number of individuals occupying the recalculated interaction area. For the full details on this procedure for correcting for edge effects, see Ralph (2021; https://petrelharp.github.io/circle_rectangle_intersection/circle_rectangle_intersection.html).

Each simulated individual was diploid (2*n*) with a haploid genome size of 1000 Mb, a recombination rate of 10^–9^, and mutation rate 10^–8^. These rates ensure ~ 1 recombination event on average per gamete and ~ 10 new mutations per gamete. During the simulation, SLiM tracked the local ancestry of each recombination breakpoint interval for all individuals via tree sequence recording^31^. In addition, we utilized SLiM’s ability to store the full pedigree of all individuals, allowing us to estimate an average of Wright’s coefficient of relatedness^73^. We did so by randomly sampling 50 individuals each generation, estimating their pedigree relatedness, and then estimating average sampled relatedness as: *F*_*r*_ = (*r* – *n*)/*n* where *r* is the sum of all values in the relatedness matrix and *n* is the sample size. Finally, we recorded mean and max age (in timesteps) of the population for each timestep, as well as recorded the number of offspring and fitness of individuals, throughout the simulation. Specific SLiM recipes for each contraction model are available at https://github.com/hancockzb.

### Analysis

Tree-sequences produced from SLiM were imported into Python. Haplotypes with multiple ancestors (i.e., coalescence had not yet occurred during the SLiM simulation) were “recapitated” using *pyslim*^31^. Mutations were then added to the trees via *msprime*^74^. Tree-sequences were then subset by time, with groups corresponding to 100 randomly selected individuals living 50 timesteps after each contraction event. For each group, we measured individual as well as mean group heterozygosity. For ease of comparison, we evaluated the decrease in mean group heterozygosity relative to the pre-contraction mean across each contraction interval, as well as 400 timesteps after the final contraction. To determine if mean group heterozygosity was significantly less post-contraction, we performed a pairwise Wilcoxon test in the R platform^75^. We used nonparametric tests throughout due to the data violating the assumptions of normality (Shapiro–Wilkes test, *p* < 4.1e−13).

To determine how spatial sampling schemes impacted our interpretation of the consequences of range contractions, we sampled 50 individuals each from 4–5 groups alive in the final generation from specific locations in the remaining range (“topleft,” “topright,” bottomleft,” “bottomright,” “center” for shrinkage and “top,” “uppermiddle,” “lowermiddle,” “lower” for amputation) and compared them to random samples of 50 individuals from the population prior to the contraction (“ancient”). The fragmentation scenario had 5 groups (“topleft,” “topright,” bottomleft,” “bottomright,” “ancient”) because there were no individuals in the center of the range post-contraction. We computed pairwise nucleotide divergence (π_12_) between each group, as well as nucleotide diversity within groups. In addition, we calculated pairwise *F*_ST_ for each group as

$${F}_{ST}=1-\frac{2\left({\pi }_{1}+{\pi }_{2}\right)}{{\pi }_{1}+2{\pi }_{12}+{\pi }_{2}},$$

where π_1_ and π_2_ is the nucleotide diversity within group 1 and 2, respectively.

To evaluate how patterns of spatial ancestry were impacted by different range contraction patterns, we randomly sampled four individuals alive 100 timesteps after the final contraction. Next, we calculated the relative genomic contribution of all ancestors living in this timestep (i.e., direct parents, grandparents, etc.). We then compare the spatial distribution of ancestors in our sampled timestep to a time-slice 50 timesteps prior to the initial contraction. Again, we calculated the genomic contribution to our four post-contraction sampled individuals from all ancestors living during the pre-contraction time-slice and plot their locations in space.

## Supplementary Information

Supplementary Information.

## Data Availability

Code for all models and analyses can be found at https://github.com/hancockzb**.**
