# Supplementary material for: Genetic and demographic consequences of range contraction patterns during biological annihilation
Source: Sci Rep. 2023 Jan 30;13:1691. doi: 10.1038/s41598-023-28927-z (PMC9886963; doi:10.1038/s41598-023-28927-z)
Supplement: Supplementary file 1 — Supplementary Information. [file 41598_2023_28927_MOESM1_ESM.docx]

Supplementary Material

**Tables**

Table S1. Summarization of post-contraction sampling results relative to the pre-contraction population for each of three simulated patterns of range contraction: shrinkage, amputation, and fragmentation.

| **Model** | **Percent range lost** | **Relative fitness** | **Population density** | **Genetic diversity (π)** | **Spatial ancestry** |
| --- | --- | --- | --- | --- | --- |
| Shrinkage | 88% | Highest in the center, decreases towards edges | Concentrated in the center of the range | Maintains high relative and absolute π | Approximates random distribution |
| Amputation | 87% | Highest in center, decreases north-south | Spread thinly across the range | Lower overall; relative π around mean in the center | Strongly skewed in the direction of the extinction front |
| Fragmentation | 86% | Highest around surviving deme centers, decreases along edges | Concentrated in center of surviving demes | Lowest overall; no individuals maintain π near mean | Skewed towards location of surviving deme; higher density demes with greater spatial spread |

Table S2. Pairwise nucleotide divergence values (π_12_) from post-contraction sampling locations for the simulated shrinkage pattern.

|  | Bottomleft | Topleft | Bottomright | Topright | Center | Ancient |
| --- | --- | --- | --- | --- | --- | --- |
| Bottomleft | 0.0001452 |  |  |  |  |  |
| Topleft | 0.0001973 | 0.0001421 |  |  |  |  |
| Bottomright | 0.0002017 | 0.0002131 | 0.0001892 |  |  |  |
| Topright | 0.0002149 | 0.0001996 | 0.0002073 | 0.0001719 |  |  |
| Center | 0.0001883 | 0.0002035 | 0.0001974 | 0.0002057 | 0.0001837 |  |
| Ancient | 0.0002648 | 0.0002609 | 0.0002644 | 0.0002626 | 0.0002168 | 0.0002543 |

Table S3. Pairwise F_ST_ values from post-contraction sampling locations for the simulated shrinkage pattern.

|  | Bottomleft | Topleft | Bottomright | Topright | Center | Ancient |
| --- | --- | --- | --- | --- | --- | --- |
| Bottomleft |  |  |  |  |  |  |
| Topleft | 0.1575 |  |  |  |  |  |
| Bottomright | 0.0935 | 0.1252 |  |  |  |  |
| Topright | 0.1509 | 0.1194 | 0.0689 |  |  |  |
| Center | 0.1108 | 0.0285 | 0.0726 | 0.0726 |  |  |
| Ancient | 0.1400 | 0.1366 | 0.0878 | 0.104 | 0.0926 |  |

Table S4. Pairwise nucleotide divergence values (π_12_) from post-contraction sampling locations for the simulated amputation pattern.

|  | Top | Uppermiddle | Middle | Lowermiddle | Lower | Ancient |
| --- | --- | --- | --- | --- | --- | --- |
| Top | 0.0001246 |  |  |  |  |  |
| Uppermiddle | 0.0001889 | 0.0001612 |  |  |  |  |
| Middle | 0.0002067 | 0.0001740 | 0.0001595 |  |  |  |
| Lowermiddle | 0.0002445 | 0.0002113 | 0.0001959 | 0.0001571 |  |  |
| Lower | 0.0002604 | 0.0002319 | 0.0002192 | 0.000165 | 0.0001056 |  |
| Ancient | 0.0002716 | 0.0002697 | 0.0002698 | 0.0002687 | 0.0002660 | 0.0002629 |

Table S5. Pairwise *F_ST_* values from post-contraction sampling locations for the simulated amputation pattern.

|  | Top | Uppermiddle | Middle | Lowermiddle | Lower | Ancient |
| --- | --- | --- | --- | --- | --- | --- |
| Top |  |  |  |  |  |  |
| Uppermiddle | 0.1386 |  |  |  |  |  |
| Middle | 0.1856 | 0.0407 |  |  |  |  |
| Lowermiddle | 0.2690 | 0.1408 | 0.1061 |  |  |  |
| Lower | 0.3871 | 0.2697 | 0.2463 | 0.1135 |  |  |
| Ancient | 0.1674 | 0.1197 | 0.1219 | 0.1226 | 0.1816 |  |

Table S6. Pairwise nucleotide divergence values (π_12_) from post-contraction sampling locations for the simulated fragmentation pattern.

|  | Topleft | Topright | Bottomleft | Bottomright | Ancient |
| --- | --- | --- | --- | --- | --- |
| Topleft | 0.0001047 |  |  |  |  |
| Topright | 0.0002765 | 0.0000238 |  |  |  |
| Bottomleft | 0.0002781 | 0.0002870 | 0.0001037 |  |  |
| Bottomright | 0.0002790 | 0.0002827 | 0.0002769 | 0.0000825 |  |
| Ancient | 0.0002653 | 0.0002686 | 0.0002625 | 0.0002681 | 0.0002585 |

Table S7. Pairwise *F_ST_* values from post-contraction sampling locations for the simulated fragmentation pattern.

|  | Topleft | Topright | Bottomleft | Bottomright | Ancient |
| --- | --- | --- | --- | --- | --- |
| Topleft |  |  |  |  |  |
| Topright | 0.6229 |  |  |  |  |
| Bottomleft | 0.4549 | 0.6366 |  |  |  |
| Bottomright | 0.4976 | 0.6837 | 0.4969 |  |  |
| Ancient | 0.1872 | 0.3111 | 0.1835 | 0.2226 |  |

**Figures**


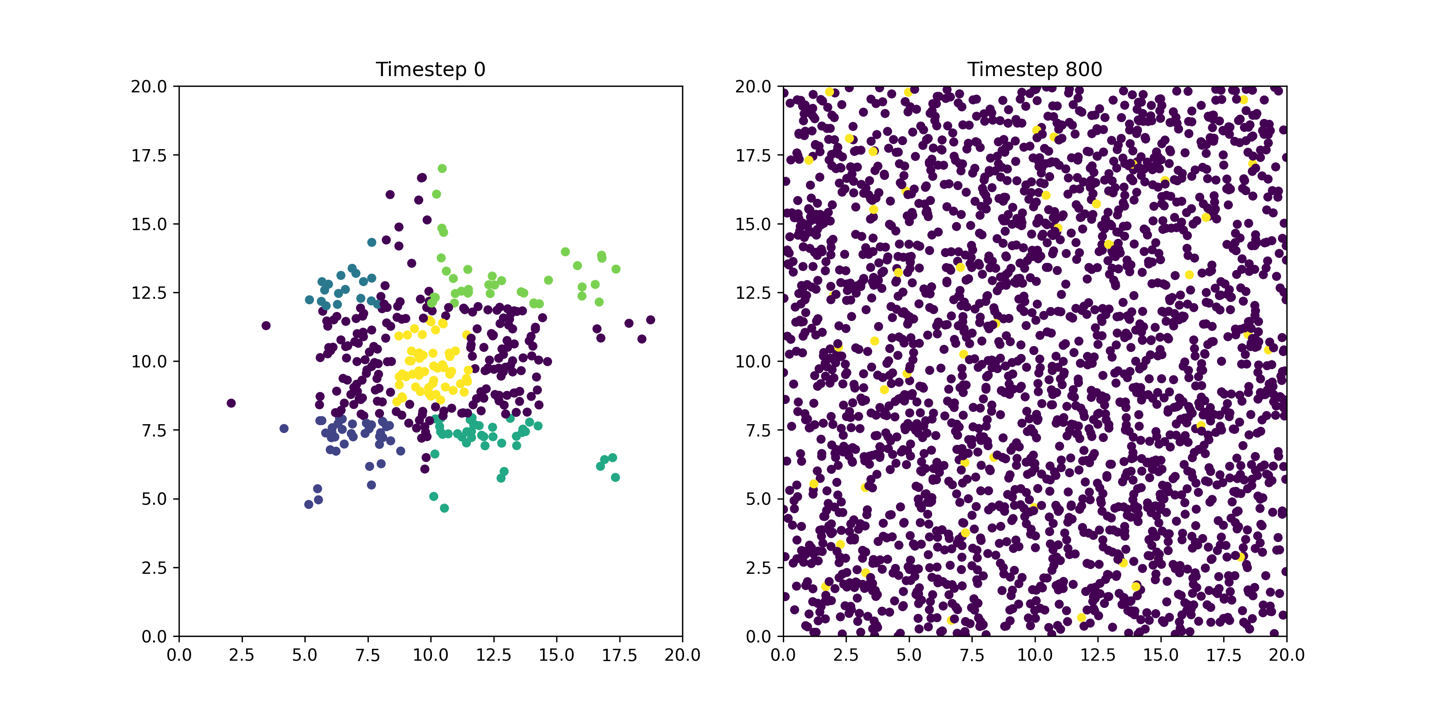


Figure S1. Pre- and post-contraction spatial sampling locations for the simulated shrinkage pattern.


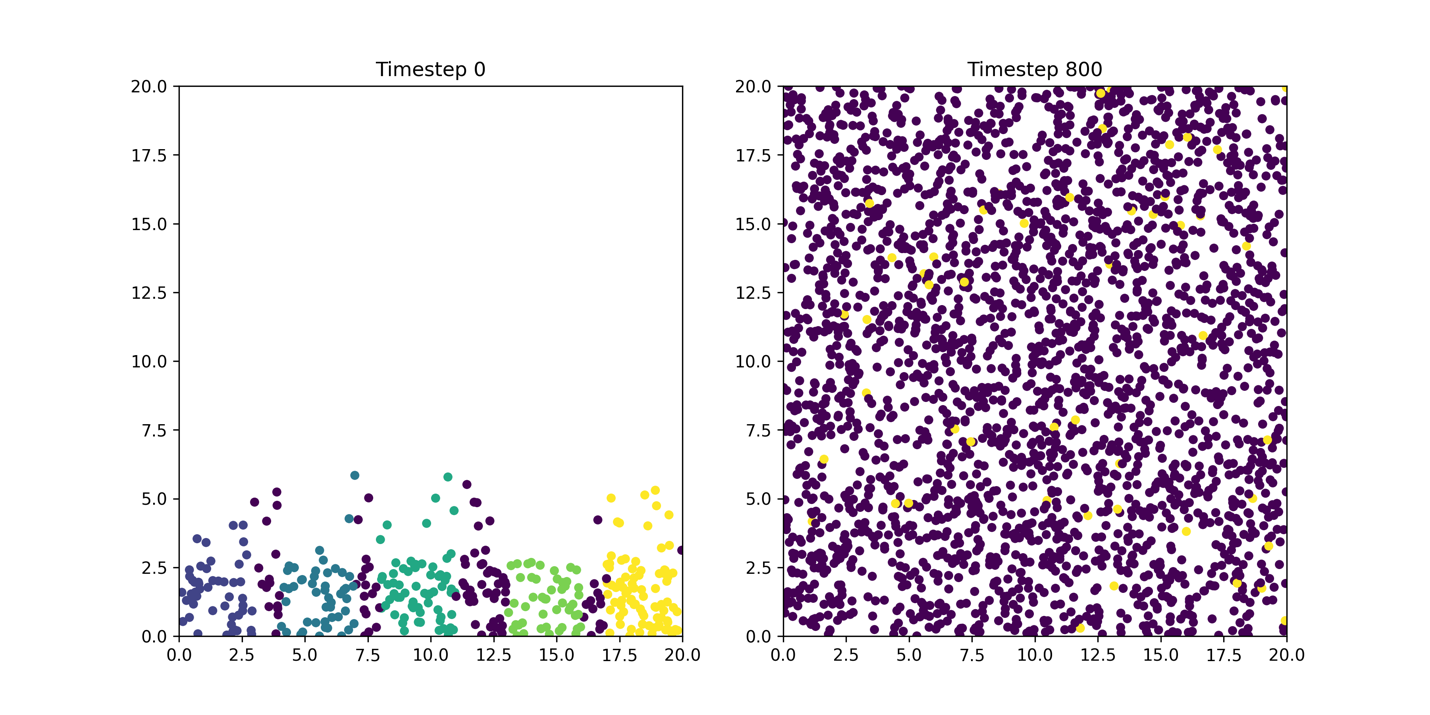


Figure S2. Pre- and post-contraction spatial sampling locations for the simulated amputation pattern.


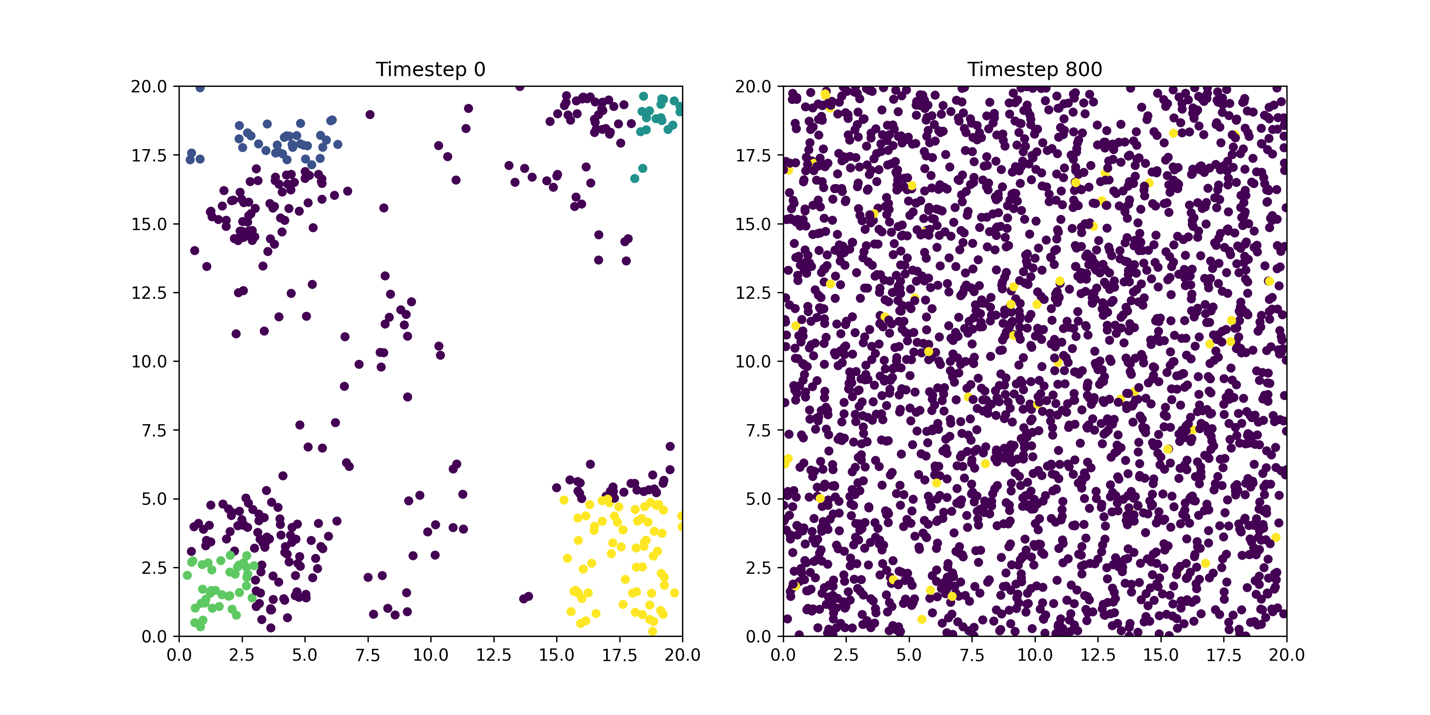


Figure S3. Pre- and post-contraction spatial sampling locations for the simulated fragmentation pattern.


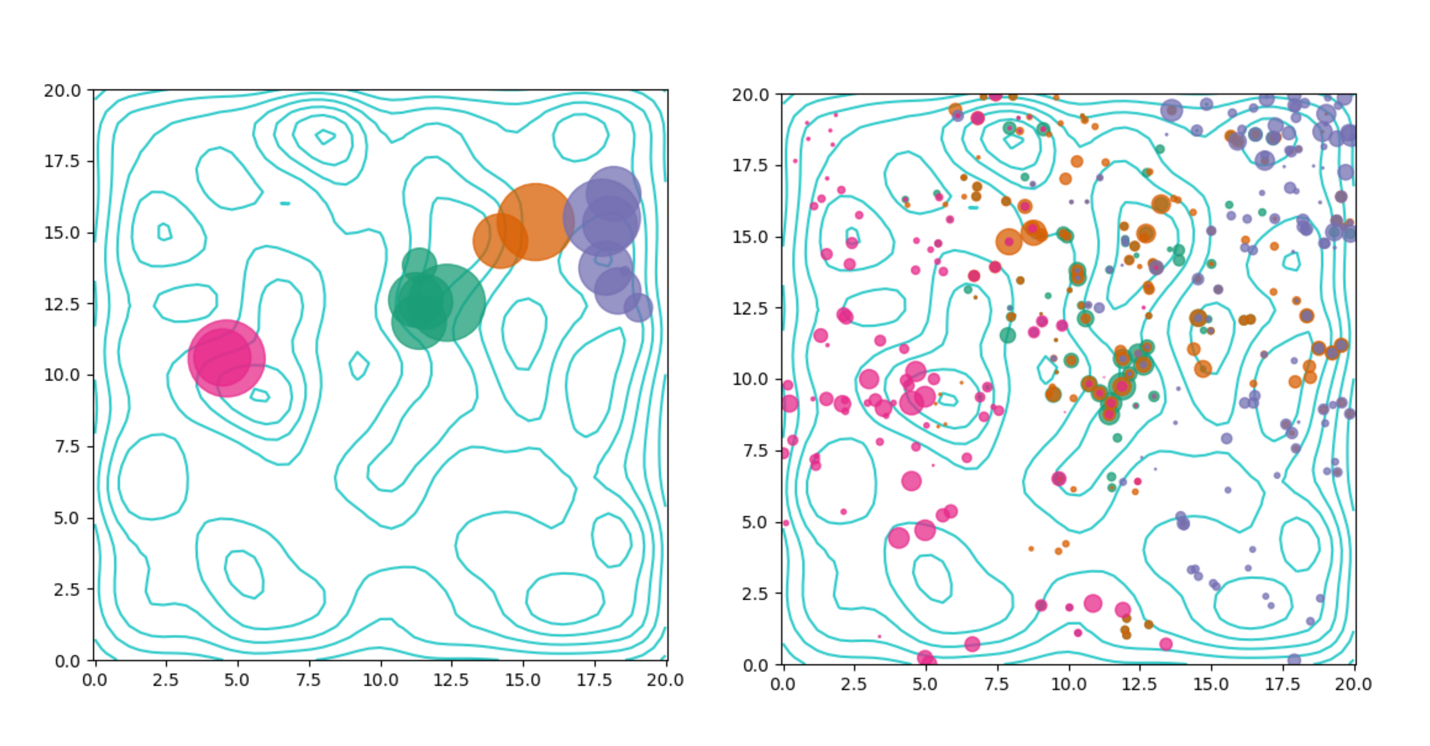


Figure S4. Spread of spatial ancestry for the randomly generated fragmentation contraction pattern.


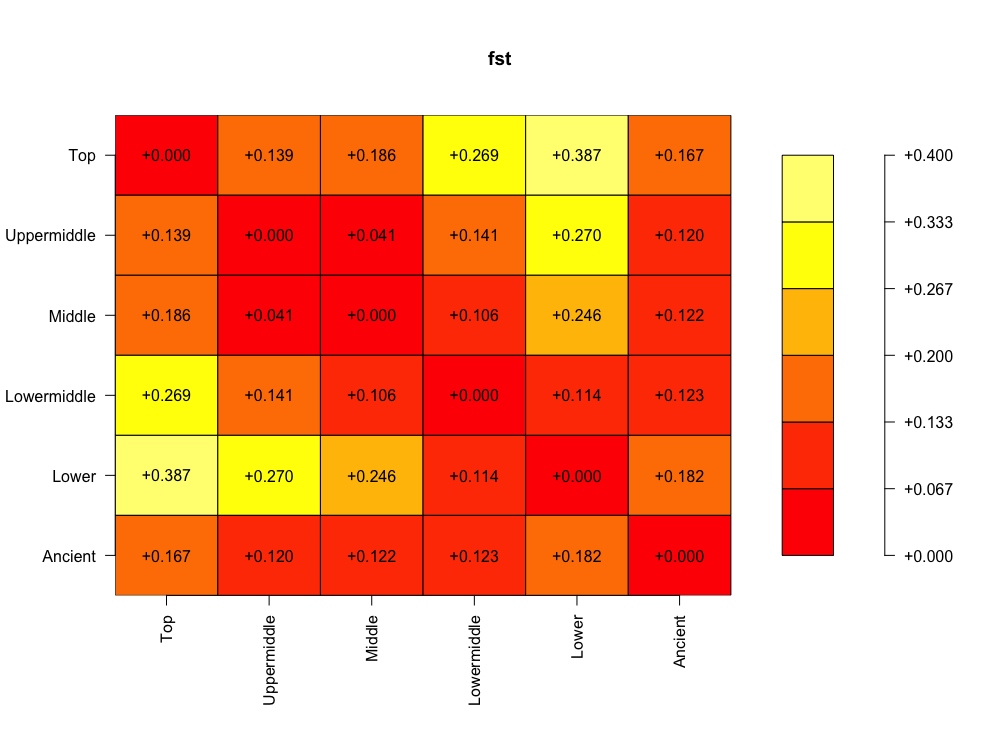


Figure S5. Colored pairwise *F_ST_* matrix for the simulated amputation pattern.
